# Transcriptomic characterization of the histopathological growth patterns in breast cancer liver metastases

**DOI:** 10.1007/s10585-024-10279-1

**Published:** 2024-03-29

**Authors:** Sophia Leduc, Ha-Linh Nguyen, François Richard, Gitte Zels, Amena Mahdami, Maxim De Schepper, Marion Maetens, Anirudh Pabba, Joris Jaekers, Emily Latacz, Ali Bohlok, Evy Vanderheyden, Thomas Van Brussel, Bram Boeckx, Rogier Schepers, Diether Lambrechts, Luc Dirix, Denis Larsimont, Sophie Vankerckhove, Valerio Lucidi, Baki Topal, Imane Bachir, Vincent Donckier, Giuseppe Floris, Peter Vermeulen, Christine Desmedt

**Affiliations:** 1 Laboratory for Translational Breast Cancer Research, Department of Oncology, KU Leuven, Herestraat 49, box 810, Leuven, 3000 Belgium; 2 Department of Pathology, University Hospitals Leuven, Leuven, Belgium; 3 Department of Visceral Surgery, University Hospitals Leuven, KU Leuven, Leuven, Belgium; 4 Translational Cancer Research Unit, GZA Hospitals Antwerp, Antwerp, Belgium; 5 Department of Surgical Oncology, Institut Jules Bordet, Université Libre de Bruxelles, Brussels, Belgium; 6 Laboratory of Translational Genetics, VIB-KU Leuven, Leuven, Belgium; 7 Department of Anatomopathology, Institut Jules Bordet, Brussels, Belgium; 8 Department of Abdominal Surgery, Erasme Hospital, Université Libre de Bruxelles, Brussels, Belgium; 9 Department of Anesthesiology, Institut Jules Bordet, Université Libre de Bruxelles, Brussels, Belgium; 10 Department of Imaging and Pathology, Laboratory of Translational Cell & Tissue Research and University Hospitals Leuven, KU Leuven, Leuven, Belgium

**Keywords:** Metastatic breast cancer, Liver metastasis, Histopathological growth pattern, Transcriptomics

## Abstract

Metastatic breast cancer (mBC) remains incurable and liver metastases (LM) are observed in approximately 50% of all patients with mBC. In some cases, surgical resection of breast cancer liver metastases (BCLM) is associated with prolonged survival. However, there are currently no validated marker to identify these patients. The interactions between the metastatic cancer cells and the liver microenvironment result in two main histopathological growth patterns (HGP): replacement (r-HGP), characterized by a direct contact between the cancer cells and the hepatocytes, and desmoplastic (d-HGP), in which a fibrous rim surrounds the tumor cells. In patients who underwent resection of BCLM, the r-HGP is associated with a worse postoperative prognosis than the d-HGP. Here, we aim at unraveling the biological differences between these HGP within ten patients presenting both HGP within the same metastasis. The transcriptomic analyses reveal overexpression of genes involved in cell cycle, DNA repair, vessel co-option and cell motility in r-HGP while angiogenesis, wound healing, and several immune processes were found overexpressed in d-HGP LM. Understanding the biology of the LM could open avenues to refine treatment of BC patients with LM.

**Supplementary Information:**

The online version contains supplementary material available at 10.1007/s10585-024-10279-1.

## Introduction

Liver is one of the most common sites for breast cancer (BC) metastases [1], and liver metastases (LM) are associated with poor prognosis [2, 3]. Currently, systemic therapy is used as the primary treatment for metastatic liver disease [3–6]. Surgical resection of LM is rarely performed in patients with mBC, however, a small fraction of patients with a localized disease benefit from this intervention [7]. As of today, there is a lack of selection criteria to distinguish patients who would benefit from local and/or systemic treatment. Our previous study demonstrated that patients with any proportion of desmoplastic growth pattern (d-HGP; i.e. at least 1% of the tumor-liver interface is desmoplastic), characterized by a fibrotic rim surrounding the tumor cells, had a better prognosis as compared to those with a ‘pure-replacement’ growth pattern (r-HGP; i.e. 100% of the tumor-liver interface is replacement), where cancer cells are in direct contact with the hepatocytes, mimicking the liver architecture [8, 9]. In a broader context, a comprehensive study of multiple types of primary cancers reported that HGP may be used as an independent marker of metastatic behavior and survival, with r-HGP reflecting a more aggressive and diffusely metastatic progression [8]. In patients with resected BCLM, 56% present with liver metastases with any proportion of d-HGP [8, 9]. This includes 45% of patients with both growth patterns at the tumor-liver interface and 11% of patients with ‘pure d-HGP’. A ‘pure r-HGP’ is observed in 44% of patients [8, 9]. Results from studies in patients with colorectal cancer LM [10] suggest that liver surgery would be particularly recommended in those patients having a LM with only a desmoplastic growth pattern, as this is associated with liver-limited disease instead of widespread systemic relapse.

Despite the clear impact of the growth patterns of LM on patient outcome, the fundamental biological mechanisms associated with the different growth patterns are, in part, still unknown. Moro et al. [11] recently demonstrated that the fibrous capsule surrounding liver metastases with a d-HGP resembles to a typical reaction of the liver to any insult: fibrosis and inflammation. The biology of the replacement growth pattern, on the other hand, remains elusive, although cancer cell motility and cell fitness have been suggested to play a role [10, 11]. In this study, we therefore aimed at exploring the biological differences between the desmoplastic and the replacement growth pattern of BCLM at the transcriptional level and by bulk RNA sequencing.

## Materials and methods

**Fig. 1:**
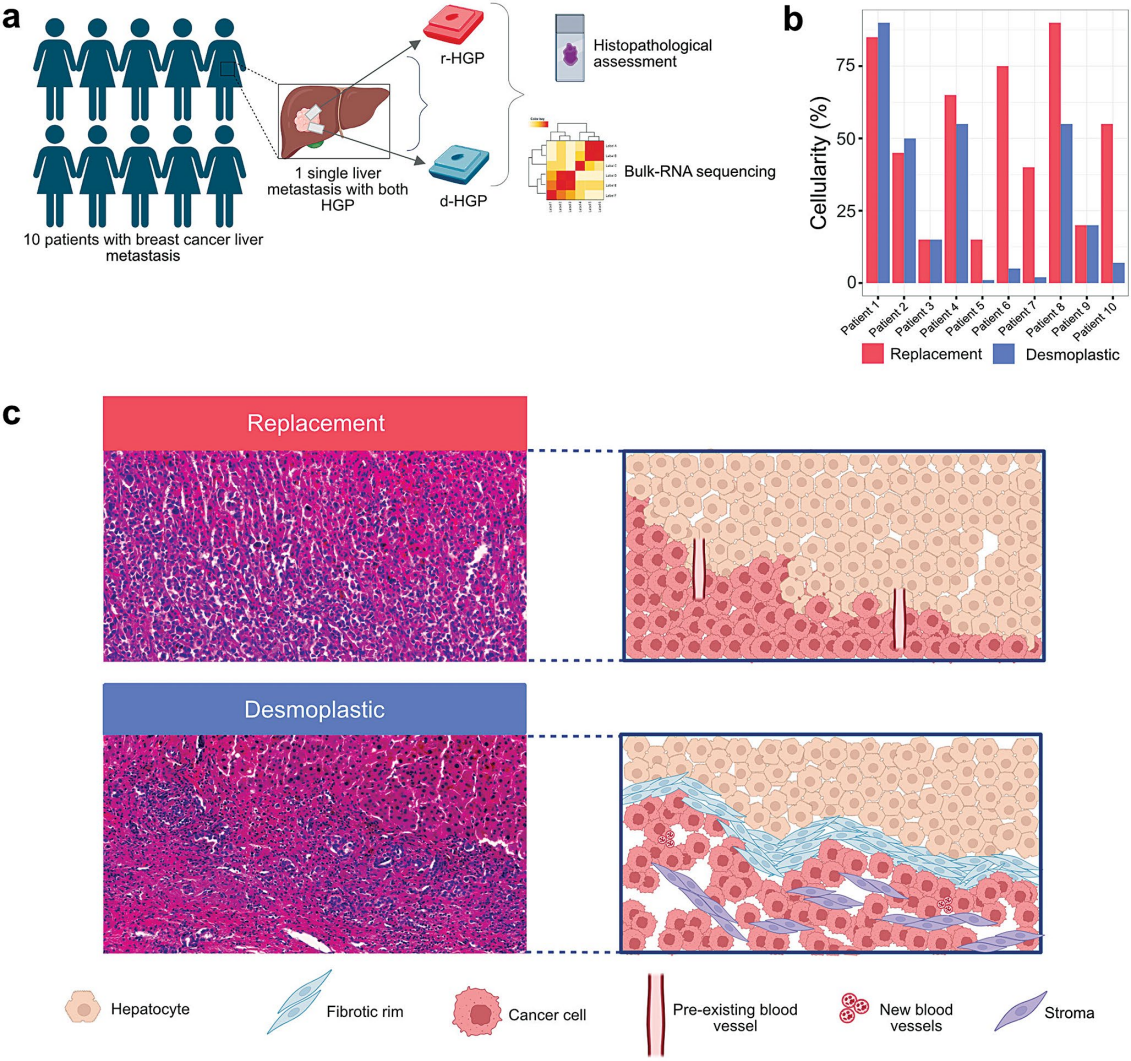
Study design and tumor cellularity assessment and Differential Gene Expression Analysis. (**a**) Study design. Inclusion of ten patients with breast cancer liver metastasis. From each liver metastasis within the same patient we have selected two samples with different HGP (r-HGP and d-HGP). Downstream analyses included histopathological assessment and bulk-RNA sequencing according to the manufacturer instructions (see Supplementary Material - ‘RNA isolation, library preparation, sequencing and data processing’ section). (**b**) Tumor cellularity. Higher tumor cellularity was observed in r-HGP BCLM in 6/10 patients. (**c**) Two haematoxylin and eosin (H&E) sections (left) and their schematic representation (right) from the same breast cancer liver metastasis. Top row: r-HGP with a direct contact between tumor cells and hepatocytes, mimicking the liver architecture, less differentiated cells and no fibrosis. Bottom row: d-HGP with a fibrous rim separating the tumor cells from the hepatocytes, more differentiated cells and fibrosis. Magnification 18.7x. r-HGP, replacement growth pattern; d-HGP, desmoplastic growth pattern. Red = r-HGP; blue = d-HGP.

We collected formalin-fixed paraffin-embedded (FFPE) samples from surgically-resected LM from 10 patients with breast cancer from 4 different Belgian hospitals (UZ Leuven, Leuven; Institut Jules Bordet, Brussels; Erasme Hospital, Brussels; GZA Ziekenhuizen, Antwerp) and for whom the two HGP were simultaneously present at the liver-tumor interface. A homogeneous set of clinico-pathological data was retrieved from local medical files. These data include but are not limited to age of the patient and menopausal status at primary diagnosis, BC histopathological parameters of the primary tumour (oestrogen receptor (ER) status, human epidermal growth factor receptor 2 (HER2) status, histological type, histological grade, laterality and multifocality), characteristics of the LM (hormone receptor status, HER2 status), the presence of extrahepatic metastasis, treatment and outcome. This study received central ethical approval (S64812; 25/03/21), Ethics Committee (EC) from UZ Leuven, Belgium. Local EC approvals were obtained, and material and data transfer agreements were set up. The vast majority (82%) of the primary tumors were identified as invasive breast carcinoma of no special type (IBC-NST) which all expressed the ER but not the HER2 (Supplementary Table 1). Of all patients, 90% (9/10) with LM were ER+/HER2^non − amp^, while 10% (1/10) were ER-/HER2^non − amp^. Interestingly, 80% (8/10) of patients developed a left-sided BC and the liver was their first site of progression. Notably, 90% (9/10) of the patients did not exhibit any extrahepatic LM but received systemic preoperative treatment. HGP was evaluated according to the international consensus guidelines [10, 12]. Each LM had to have two different FFPE blocks with a predominance (≥ 40%) of one HGP, resulting in 20 samples (Fig. 1a; Supplementary Fig. 1). RNA was extracted from the FFPE samples (Qiagen kit) [13]. The differential transcriptomic profile between the two HGP was assessed using differential gene expression analysis (DGEA) and gene set enrichment analysis (GSEA). These analyses accounted for the tumor abundance by taking as a covariate either tumor cellularity, or the Microenvironment Score (MES) derived from the deconvolution using xCell [14]. P-values presented were not formally corrected for multiple testing. More details on materials and methods are provided in the Supplementary Material.

## Results

We assessed the tumor cellularity and tumor-infiltrating lymphocyte (TIL) on hematoxylin and eosin (H&E) slides and observed that these differed according to the HGP.

Firstly, we found that in 6/10 patients, r-HGP exhibited higher tumor cellularity (median: 50%; range: 15-85%) compared to its matched d-HGP samples (median: 17%; range: 1-90%) and that two patients had the same cellularity in both samples from the same metastasis (Fig. 1b-c). Additionally, and as expected, we observed a negative correlation between pathologically assessed tumor cellularity and computationally inferred MES (Spearman’s Correlation Coefficient= -0.376; p-value = 0.102) (Supplementary Fig. 2). As gene expression levels measured by bulk-RNA sequencing are derived from all cells in the tissue fragment used for analysis, an adjustment for tumor cell content is regarded as necessary for comparison of the different samples of the experiment. The observation that both adjustment approaches (tumor cellularity and MES) yield similar results in subsequent analyses, is reassuring, suggesting that the deconvolution results are reliable.

Regarding the TIL, higher levels were observed in the d-HGP (median: 10%; range: 4-53%) as compared to the r-HGP (median: 4%; range: 1-39%) BCLM although not significantly (p-value = 0.101; Supplementary Fig. 3a). We observed the same trend for the ImmuneScore inferred by deconvolution (p-value = 0.970, Supplementary Fig. 3b). Lastly, a positive moderate correlation between the TIL and ImmuneScore was seen (Spearman’s Correlation Coefficient = 0.382, p-value = 0.096, Supplementary Fig. 3c).

**Fig. 2:**
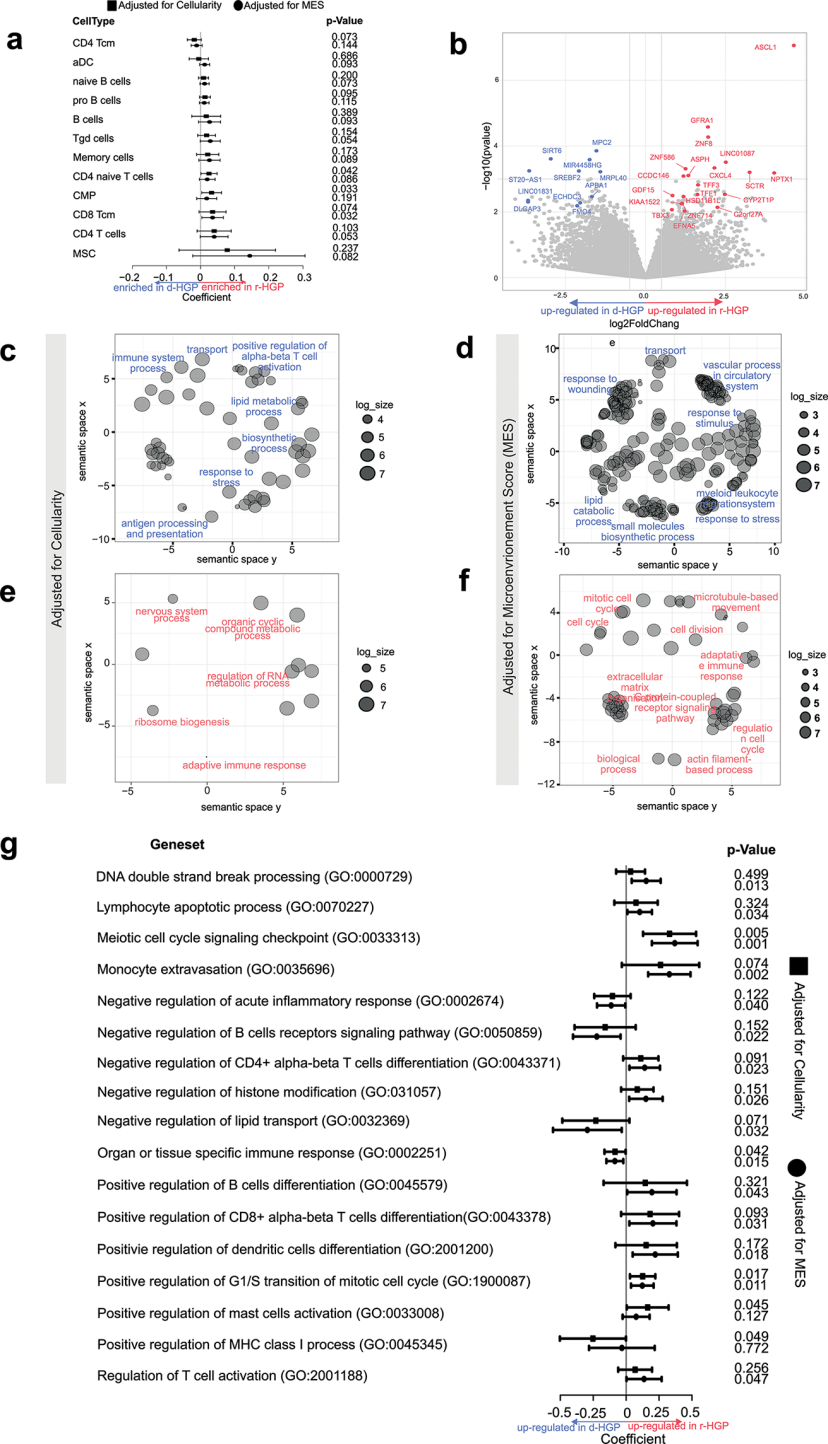
Association of immune cell types with HGP, Differential Gene Expression Analysis (DGEA) and Gene Set Expression Analysis (GSEA). (**a**) The associations were estimated by linear mixed models adjusted for the tumor cellularity and the Microenvironment Score (MES). Cell types with a p-value < 0.1 in one of the two models are shown. A positive estimate indicates a positive association with the r-HGP. Tcm = central memory T cells; aDC = activated dendritic cells; Tgd cells = gamma-delta T cells; CMP = common myeloid progenitor; MSC = mesenchymal stem cells. (**b**) Volcano Plot of DGEA. Genes with |logFC| > 0.5 and a p-value < 0.001 were highlighted and labeled. Additionally, genes with |logFC| > 0.5 and p-value < 0.01 in both analyses (tumor cellularity as covariate, and MES as covariate) were also highlighted and labeled. Genes highlighted in red were up-regulated in r-HGP, and genes highlighted in blue were up-regulated in d-HGP. **c-f.** Gene clustering. Up-regulated genes in d-HGP (top panels) and r-HGP (bottom panels) with a p-value < 0.1 were selected from each of the analyses, tumor cellularity as covariate (**c-e**), and MES as covariate (**d-f**), as input. **g**. Association of gene set enrichment and cell composition with HGP. Forestplots showing the association of the Gene Set Variation Analysis (GSVA) scores of Gene Ontology Biological Processes (GOBP) gene sets. The associations were estimated by linear mixed models adjusted for the tumor cellularity and MES. Selected gene sets (by meaning) with a p-value < 0.05 in one of the two models which are discussed in the text are shown. A positive estimate indicates a positive association with the r-HGP. d-HGP = desmoplastic growth pattern; r-HGP = replacement growth pattern

We next assessed the association between HGP and the enrichment of cell sub-populations derived from the deconvolution analysis (Fig. 2a, Supplementary Tables S2-S4, Supplementary Fig. 4). Focusing on immune cells, we observed an enrichment of several immune cell types, such as central memory CD8 T cells (CD8 TCm) (p-value = 0.074, adjusted for MES), CD4 naïve T cells (p-value = 0.042, adjusted for tumor cellularity) in r-HGP. On the other hand, only the CD4 + central memory T cells seemed to be more enriched in d-HGP (p-value = 0.073, adjusted for tumor cellularity). However, given the limitations of deconvolution these results need to be interpreted with caution.

DGEA of the 20 matched samples identified ten overexpressed genes (genes with |logFC| > 0.5 and a p-value < 0.001 were highlighted and labeled as shown in Supplementary Fig. 5) in r-HGP BCLM that were mainly associated with cell cycle and proliferation, DNA repair and nervous system (*ASCL1, GFRA1, ZNF8, ZNF586, LINC01087, CCDC146, ASPH, CXCL4, SCTR and NPTX1)* (Fig. 2b; Supplementary Fig. 5; Supplementary Tables S5-S6). In contrast, six genes known to inhibit cell proliferation and tumor growth were overexpressed in d-HGP: *SIRT6, MPC2, MIR4458HG, ST20-AS1, SREBF2, MRPL40* (Fig. 2b; Supplementary Fig. 5; Supplementary Tables S5, S6). A gene clustering analysis of all genes overexpressed in r-HGP revealed that these genes were mainly involved in regulation of the cell cycle, cell division, extracellular matrix organization, nervous system process and actin filament based-process (Fig. 2d-f, Supplementary Tables S5-S6). On the other hand, genes overexpressed in d-HGP BCLM were more associated with response to stress, immune activities and wound healing (Fig. 2c-e; Supplementary Tables S5, S6).

GSEA revealed that pathways related to DNA double-strand break (DSB) repair, histone modification, nervous system, mitosis and meiosis were enriched in r-HGP BCLM (Fig. 2g, Supplementary Tables S7, S8). Figure 2g also shows that gene sets related to the immune contexture were differentially enriched when comparing both growth patterns.

Finally, to explore some potential clinical relevance with regard to treatment targets, we compared the expression of the targets of immune checkpoint inhibitors (ICI) and antibody-drug conjugates (ADC), which are currently approved in BC or under clinical investigation in oncology, between the two growth patterns (Supplementary Figs. 6–9). Based on the literature and ongoing clinical trials, we identified 23 ICI and 56 ADC [15] targets. We observed no significant differences in expression between desmoplastic and replacement samples. However, *CD47*, *LGALS9* and *TNFRSF14* as well as *ALCAM*, *CD46*, *ERBB3*, *FN1* generally have high expression levels in our LM. It remains however to be demonstrated whether this is the case in a larger series of LM and whether this also translates into high expression levels at the protein level in the relevant cell subpopulations.

## Discussion

This is the first study characterizing transcriptomic profiles of surgically resected BCLM according to the HGP. We identified differentially expressed genes between r-HGP and d-HGP. The genes overexpressed in r-HGP BCLM were mainly associated with cell cycle regulation and proliferation, DNA repair and nervous system. Among them, *ASCL1*, a key transcription regulator that drives the axon regeneration [16], was highly overexpressed in r-HGP. Gene clustering supported these findings showing overexpression of genes involved in nervous system and actin filament-based processes, which is also related to axon guidance mechanisms used for vessel co-option in brain metastases [17], and to increase motility of cancer cells in r-HGP [18]. In d-HGP samples, we also observed overexpression of genes involved in wound healing which is associated to angiogenesis, a mechanism well-known to be associated with d-HGP [10], and genes involved in multiple immune processes. This finding supports the hypothesis made by Moro et al. that the desmoplastic rim may have arisen from a fibrotic and inflammatory response of the liver [11]. Furthermore, GSEA highlighted enrichment of gene sets related to the meiotic cell cycle in r-HGP. This is consistent with previous findings which indicate that cancer cells are able to use meiotic genes to help in the process of DSB repair [19], and therefore, potentially giving a survival advantage for the tumor cells in r-HGP BCLM. Our results are in line with those by Hu et al. [20]. , who showed that colorectal cancer liver metastases with d-HGP LM were significantly enriched in epithelial-mesenchymal transition, angiogenesis, stroma, and immune signaling pathways, while r-HGP were enriched in metabolism, cell cycle, and DNA damage repair pathways.

The study of the morphology of colorectal cancer [21–23] and BCLM [8] has shown differences in the density of immune cell infiltrates at the border of these metastases associated with the type of growth pattern. Desmoplastic LM typically have a rim of a mononuclear immune cell infiltrate positioned at the interface between fibrous tissue of the capsule and the adjacent liver tissue. In replacement-type metastases, this immune infiltrate is mostly absent. This indicates that the d-HGP and r-HGP differ in immune contexture. The results of our bulk RNA sequencing confirm these morphological observations. The differences in gene expression levels, gene set enrichment and immune cell content after deconvolution by xCell all indicate that growth patterns need to be considered in further elaborated analyses of the immune cell populations and immune activities, in order to fully understand the immune microenvironment of LM. Finally, our results showed that the r-HGP samples showed greater tumor cellularity, indicating a lower degree of differentiation and less fibrosis. This reflects the differences in prognosis and proliferation between the two HGP.

We also investigated the difference in expression of various IC and ADC markers in desmoplastic and replacement samples. Although we did not observe a significant difference, several genes appeared to be highly expressed in liver metastases and could be further investigated as potential targets.

Although the impact on patient outcome of the HGP of LM is demonstrated by many studies and independent of primary tumor types, the effect of the relative amount of each HGP in lesions with mixed, not pure, HGP remains unclear. Indeed, Moro et al. [11] showed that the amount of encapsulation, rather than the mere presence of replacement growth, affects outcome in patients with mixed HGP colorectal LM after surgery. This remains to be further investigated in larger series of patients with surgically resected BCLM.

We acknowledge the limitations of this study, its retrospective nature, the small sample size, and the fact that the samples were taken at a specific point in time, which may not reflect the dynamic nature of the HGP. Therefore, to further characterize BCM according to their growth pattern, we plan to use various single-cell and spatial omics technologies, as well as experimental models. To this end, we have initiated a prospective multicentric study, OLiver Pro (NCT05720676). In this study, patients with BCLM scheduled for surgical resection will be prospectively included and well-annotated mirrored fresh, fresh frozen and FFPE samples of the center of the liver metastasis, the tumor-liver interface, and the adjacent normal liver parenchyma will be collected. The objectives of OLiver Pro are: (1) to conduct a comprehensive clinical, histopathological and in-depth molecular characterization using single-cell and spatial omics technologies, which was not possible in the present study since we only had FFPE tissue available; and (2) to establish experimental models of BCLM in to experimentally characterize the HGP and their tumor immune microenvironment.

To conclude, our results provide preliminary information on the biological differences present in BCLM according to the HGP, with overexpression of genes involved in cell cycle, DNA repair, vascular co-option and cell motility in r-HGP and angiogenesis, wound healing and various immune processes in d-HGP.

These results, which will need to be confirmed in a larger series, contribute towards a better understanding of the mechanisms driving the HGP of BCLM. We believe that by increasing the biological knowledge, we will ultimately be able to refine the treatment decision-making and the outcome of these patients. In that context, it will also be necessary to be able to identify growth patterns before surgery is performed, for example by using medical imaging aided by a radiomics tool [24, 25].

## Electronic supplementary material

Below is the link to the electronic supplementary material.

Supplementary Material 1

Supplementary Material 2

Supplementary Material 3

Supplementary Material 4

## Data Availability

Raw sequencing reads from the RNA-seq experiments (FASTQ files) have been deposited in the European Genome-phenome Archive (EGA) under study EGAS50000000225. The clinical, histological and processed data used in this manuscript are provided via a CodeOcean capsule (see Code availability). Code availability The R code for data analyses is available in a Code Ocean capsule (10.24433/CO.4761683.v1).

## Abbreviations

BC: breast cancer
BCLM: breast cancer liver metastasis
d-HGP: desmoplastic HGP
H&E: Hematoxylin and Eosin
HGP: histopathological growth pattern
LM: liver metastasis
mBC: metastatic breast cancer
MES: microenvironment score
r-HGP: replacement HGP
