# Supplementary material for: Transcriptomic characterization of the histopathological growth patterns in breast cancer liver metastases
Source: Clin Exp Metastasis. 2024 Mar 29;41(5):699–705. doi: 10.1007/s10585-024-10279-1 (PMC11499425; doi:10.1007/s10585-024-10279-1)
Supplement: Supplementary file 1 — Supplementary Material 1 [file 10585_2024_10279_MOESM1_ESM.docx]

**Supplementary Table 1: Clinico-pathological characteristics of the patients.**

| **Clinicopathological characteristics of the primary disease** | | |
| --- | --- | --- |
|  |  | **N = 10 (%)** |
| **Menopausal status (post- vs pre-menopausal)** | |  |
|  | post-menopausal | 1 (12.5) |
|  | pre-menopausal | 7 (87.5) |
|  | Missing | 2 |
| **Age (>50 vs ≤50 years)** | |  |
|  | ≤ 50 | 8 (80.0) |
|  | >50 | 2 (20.0) |
| **cT (>1 vs 1)** | |  |
|  | 1 | 1 (16.7) |
|  | 2 | 5 (83.3) |
|  | Missing | 4 |
| **cN (≥1 vs 0)** | |  |
|  | 0 | 2 (25.0) |
|  | 1 | 4 (75.0) |
|  | Missing | 4 |
| **cM (1 vs 0)** | |  |
|  | 0 | 7 (70.0) |
|  | 1 | 3 (30.0) |
| **pN (1 vs 0)** | |  |
|  | 0 | 1 (33.3) |
|  | 1 | 2 (66.7) |
|  | Missing | 7 |
| **Histological subtype (ILC vs NST)** | |  |
|  | invasive ductal adenocarcinoma (NST) | 8 (80.0) |
|  | invasive lobular adenocarcinoma (ILC) | 2 (20.0) |
| **Histological grade (2 and 3 vs 1)** | |  |
|  | 1 | 1 (14.8) |
|  | 2 | 6 (85.2) |
|  | Missing | 3 |
| **Laterality (right vs left)** | |  |
|  | left | 8 (80.0) |
|  | right | 2 (20.0) |
| **ER-status (positive vs negative)** | |  |
|  | positive | 10 (100.0) |
| **HER2-status (amplified vs non-amplified)** | |  |
|  | non-amplified | 8 (88.9) |
|  | Missing | 2 |
| **Neoadjuvant chemotherapy (yes vs no)** | |  |
|  | no | 6 (60.0) |
|  | yes | 4 (40.0) |
| **Clinicopathological characteristics of the liver metastasis** | | |
| **ER-status (positive vs negative)** | |  |
|  | negative | 1 (10.0) |
|  | positive | 9 (90.0) |
| **HER2-status (amplified vs non-amplified)** | |  |
|  | non-amplified | 10 (90.9) |
| **Extrahepatic metastasis (yes vs no)** | |  |
|  | no | 9 (90.0) |
|  | yes | 1 (10.0) |
| **Time between BC diagnosis and liver surgery (continuous)** | |  |
|  | <1year | 2 (20.0) |
|  | ≥2years | 8 (80.0) |
| **Systemic preoperative treatment before liver surgery (yes vs no)** | |  |
|  | no | 1 (10.0) |
|  | yes | 9 (90.0) |
| **First site of progression (liver vs other)** | |  |
|  | liver (only) | 8 (80.0) |
|  | other | 2 (20.0) |

**Abbreviations**: c= clinical; T= tumor; N= lymph nodes; M= metastasis; p= pathological; ILC= invasive lobular breast cancer; NST= no special type; ER= estrogen receptor; PR= progesterone receptor; HER2= human epidermal growth factor 2; BC = breast cancer.
