# Supplementary material for: Transcriptomic characterization of the histopathological growth patterns in breast cancer liver metastases
Source: Clin Exp Metastasis. 2024 Mar 29;41(5):699–705. doi: 10.1007/s10585-024-10279-1 (PMC11499425; doi:10.1007/s10585-024-10279-1)
Supplement: Supplementary file 2 — Supplementary Material 2 [file 10585_2024_10279_MOESM2_ESM.docx]

## **Supplementary Figures to *“Transcriptomic characterization of the histopathological growth patterns in breast cancer liver metastases”* by Sophia Leduc*, Ha Linh Nguyen* *et al.***


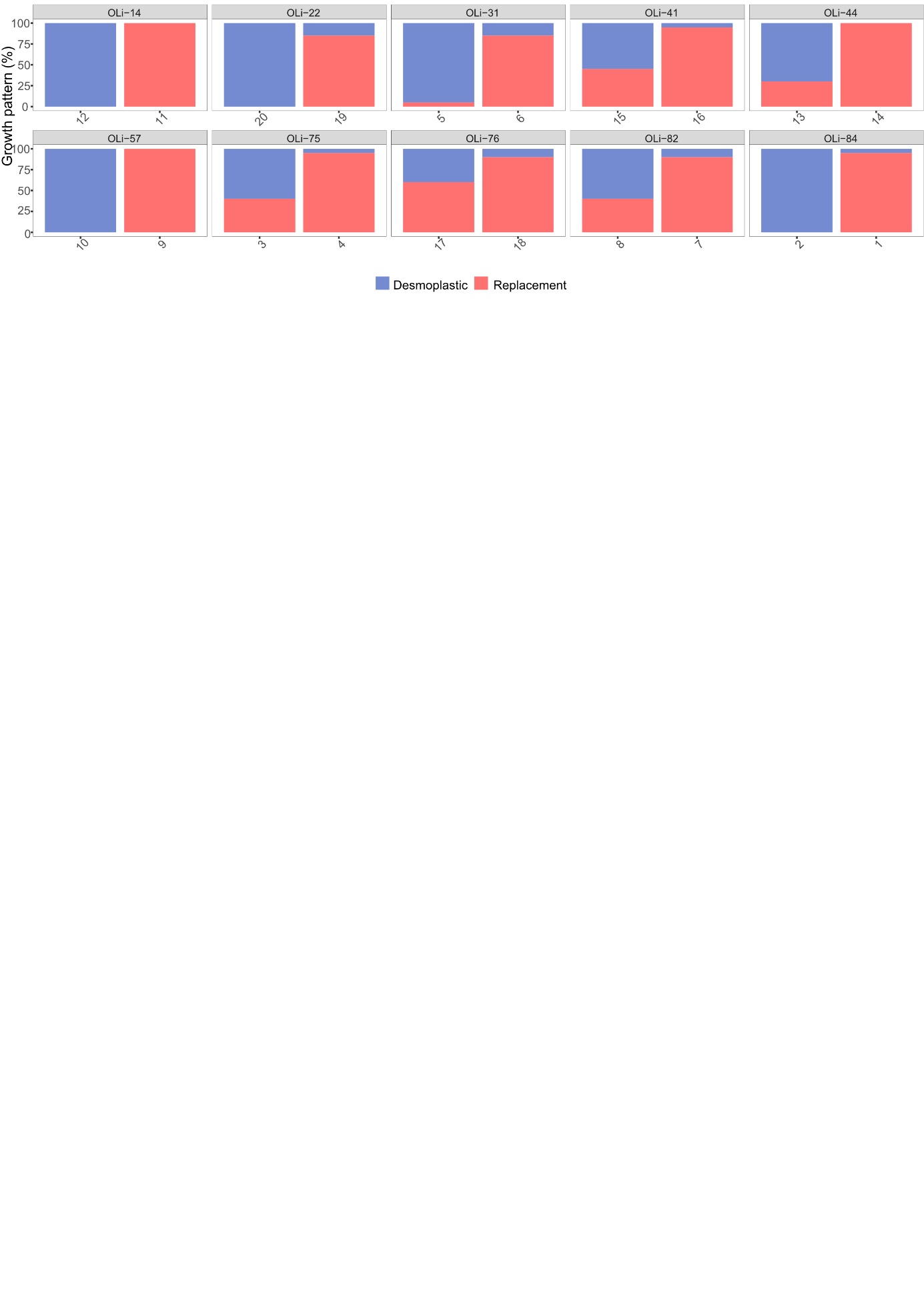


**Supplementary Figure 1: HGP distribution.** For each patient, we selected two slides from the metastasis with more than 30% of each growth pattern.


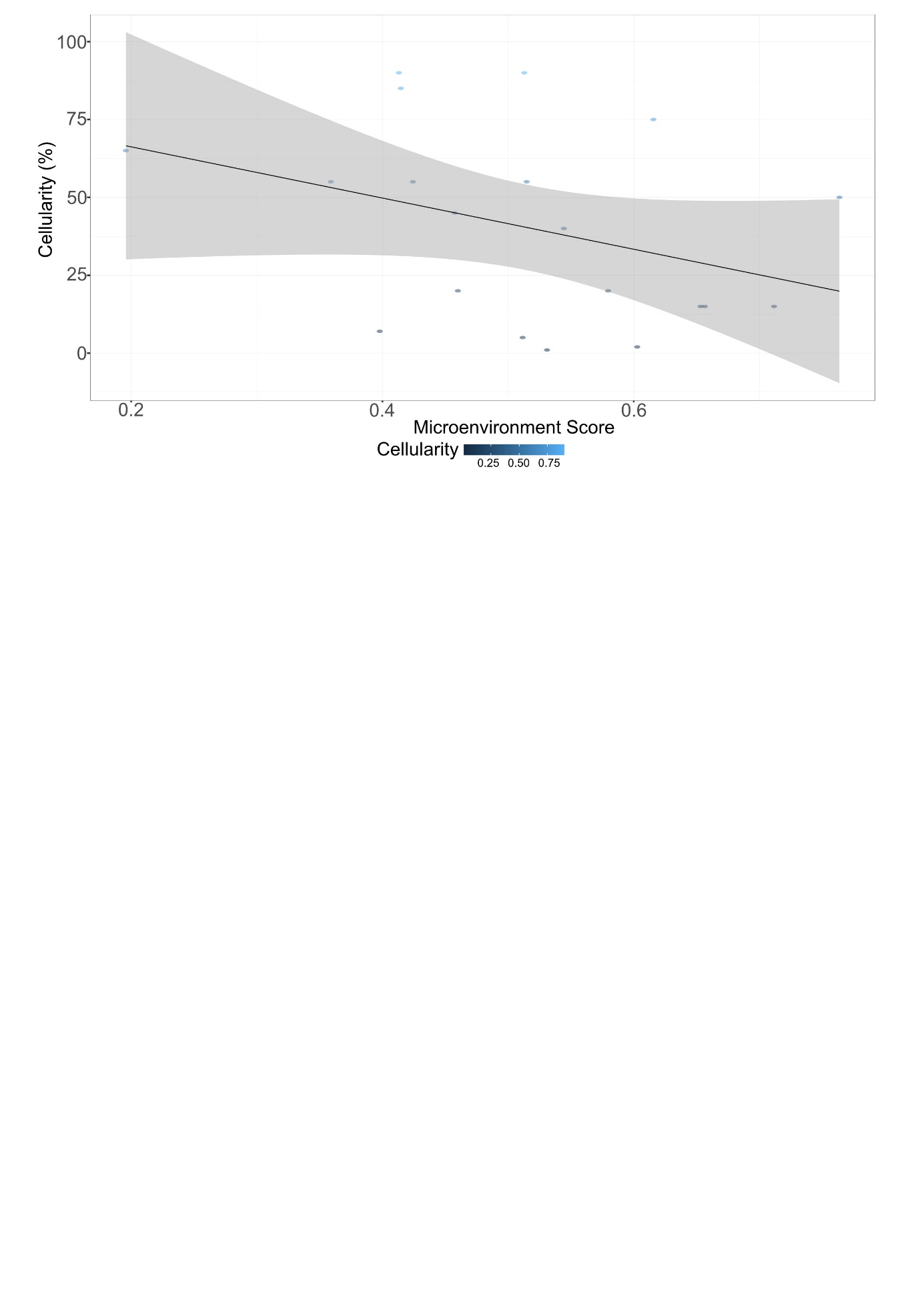


**Supplementary Figure 2: Correlation between Tumor Cellularity and Microenvironment Score (MES)** (Spearman correlation coefficient= -0.376; p-value=0.102).


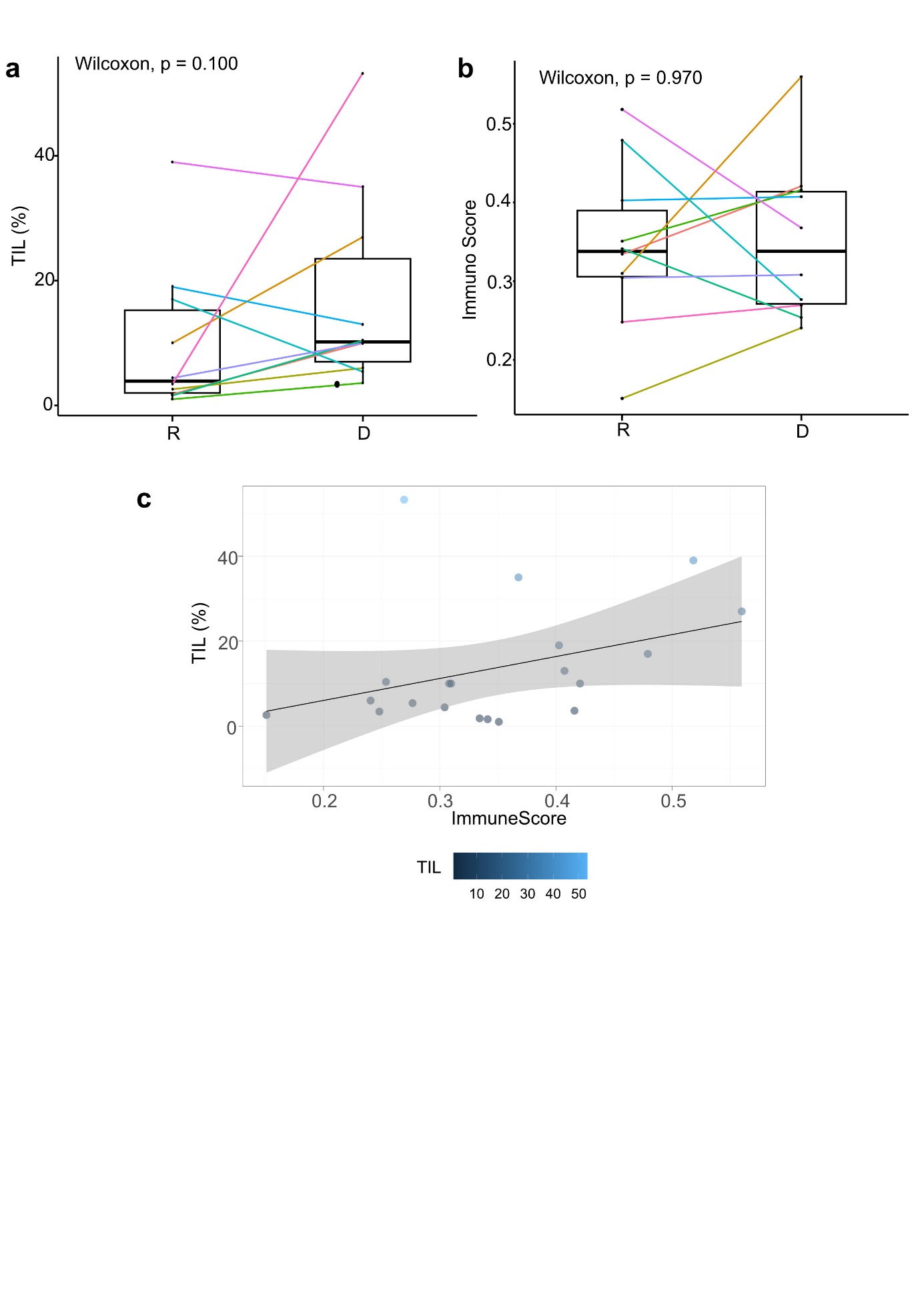


**Supplementary Figure 3: TIL distribution and correlation between TIL and ImmunoScore. a.** TIL scores according to the HGP (Wilcoxon p-value=0.100)**. b.** Immune Score according to the HGP (Wilcoxon p-value=0.970)**. c.** Correlation between the TIL score and the Immune Score calculated by xCell (Spearman correlation coefficient = 0.382; p-value = 0.096). Abbreviations: R= Replacement; D= Desmoplastic; One color per patient ID.


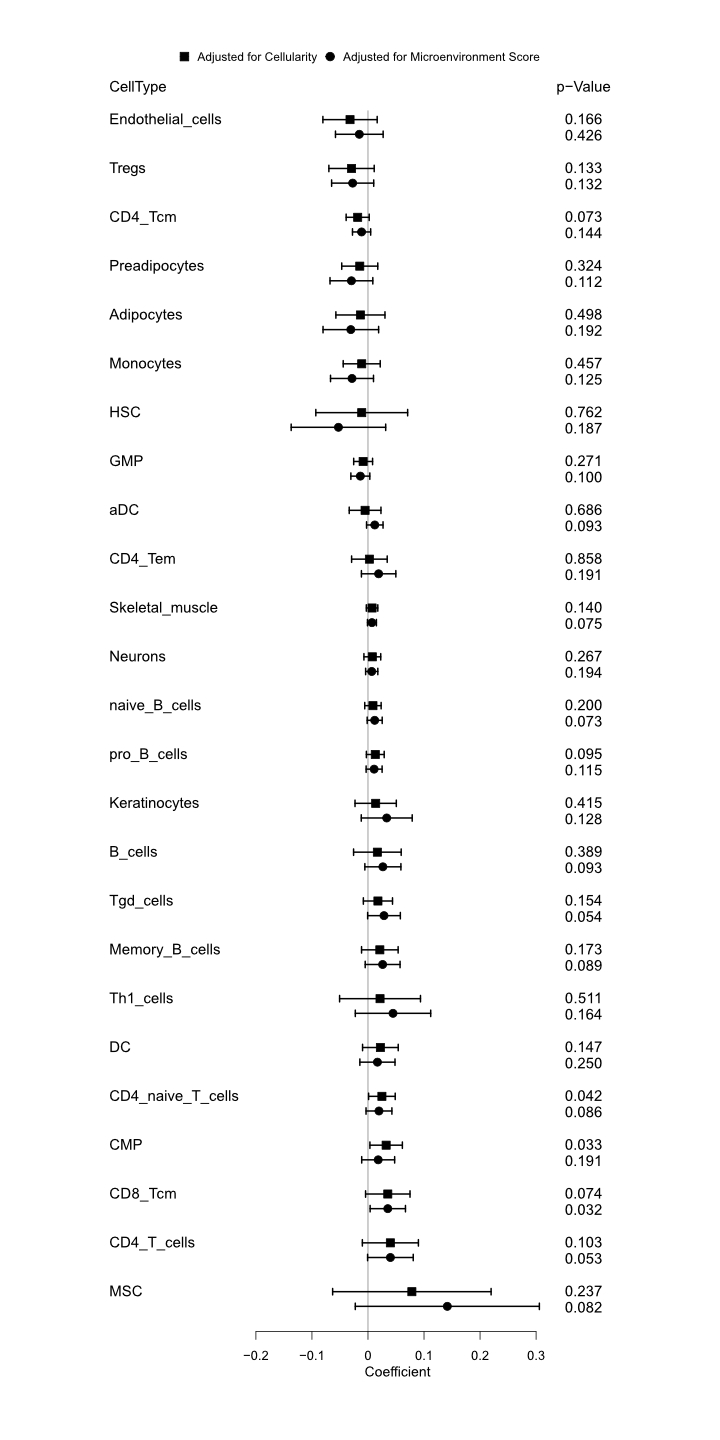


**Supplementary Figure 4:** **Association of immune cell types with HGP.** The associations were estimated by linear mixed models adjusted for the tumor cellularity and the Microenvironment Score (MES). Cell types with a p-value < 0.2 in one of the two models are shown. A positive estimate indicates a positive association with the r-HGP. Abbreviations: Tcm = central memory T cells; HSC =Hematopoietic stem cells; GMP = Granulocyte-macrophage progenitors; Th1= Type 1 T-helper cells; DC= dendritic cells; aDC = activated dendritic cells; Tgd cells = gamma-delta T cells; CMP = common myeloid progenitor; MSC = mesenchymal stem cells.

**­­­**


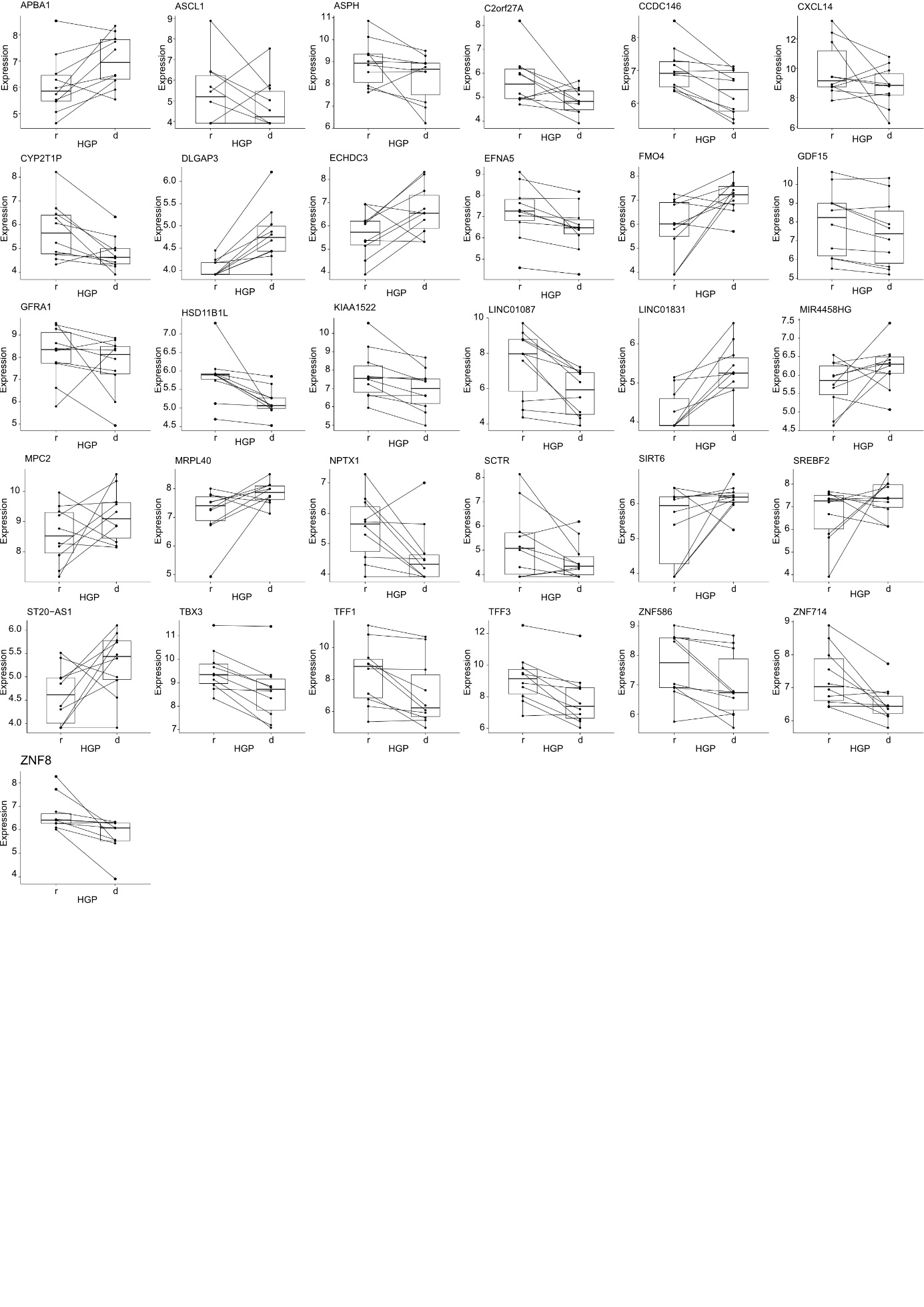


**Supplementary Figure 5: Normalized expression of the 31 differential genes according to HGP detected by differential gene expression analyses.** Genes with |logFC| > 0.5 and a p-value < 0.001 in analysis with tumor cellularity as covariate were shown. Additionally, genes with |logFC| > 0.5 and p-value < 0.01 in both analyses (tumor cellularity as covariate, and MES as covariate) were also shown. Abbreviations: r= replacement; d= desmoplastic; HGP= histopathological growth pattern.


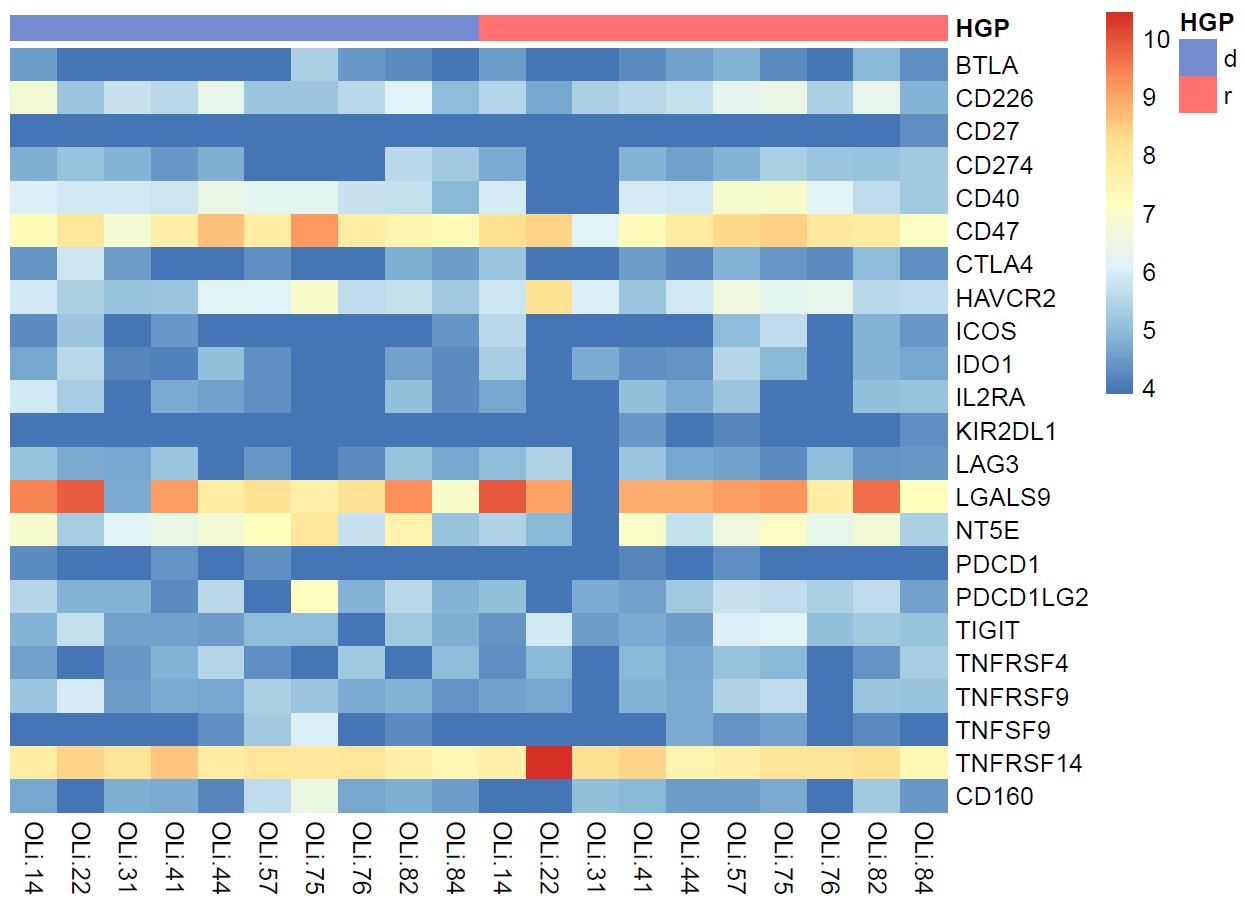


**Supplementary Figure 6: Heatmap of Immune Checkpoint (IC) markers expression**. No difference in the expression of IC genes between d-HGP and r-HGP samples. *CD47*, *LGALS9* and *TNFRSF14* are the top three highly expressed genes in all samples.

**
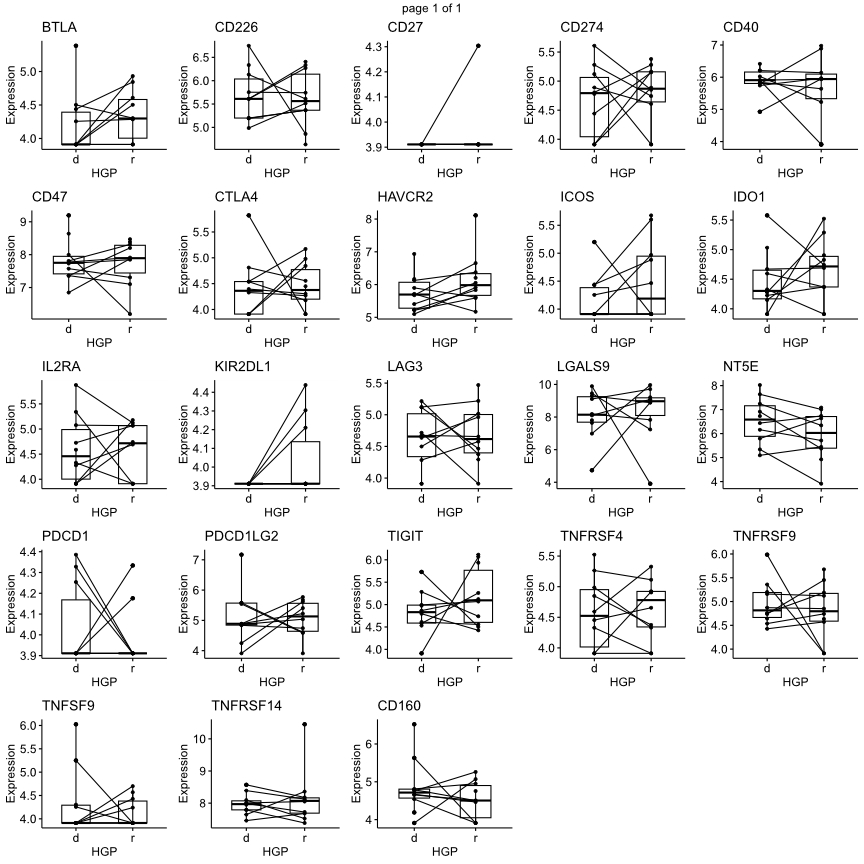
**

**Supplementary Figure 7: Normalized expression of the 23 IC genes according to HGP**. Abbreviations: r= replacement; d= desmoplastic; HGP= histopathological growth pattern.

**
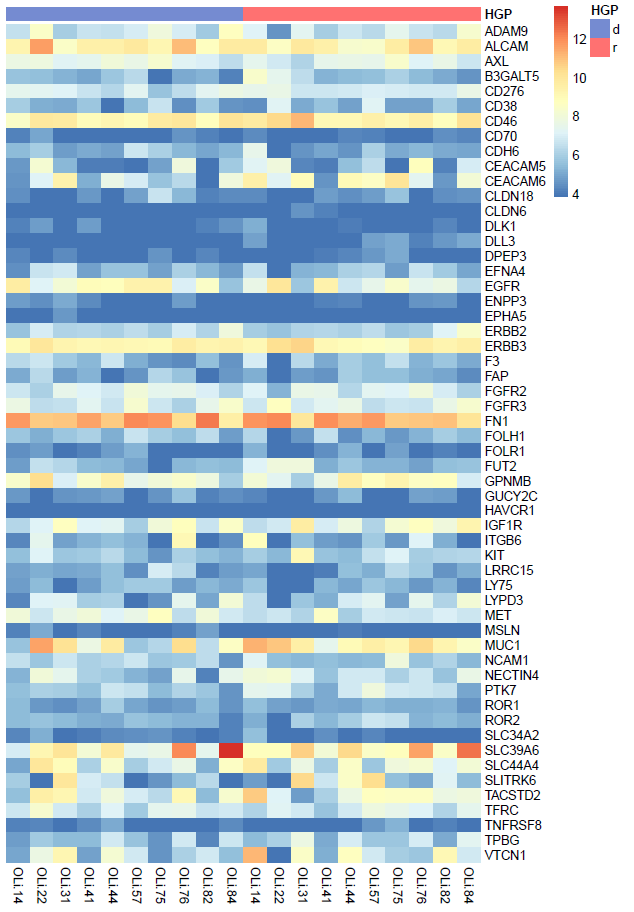
**

**Supplementary Figure 8: Heatmap of Antibody Drug Conjugate (ADC) markers expression**. No differences in the expression of ADC gene between d-HGP and r-HGP samples. *FN1*, *ALCAM* and *CD46* are the top three highly expressed genes in all samples.


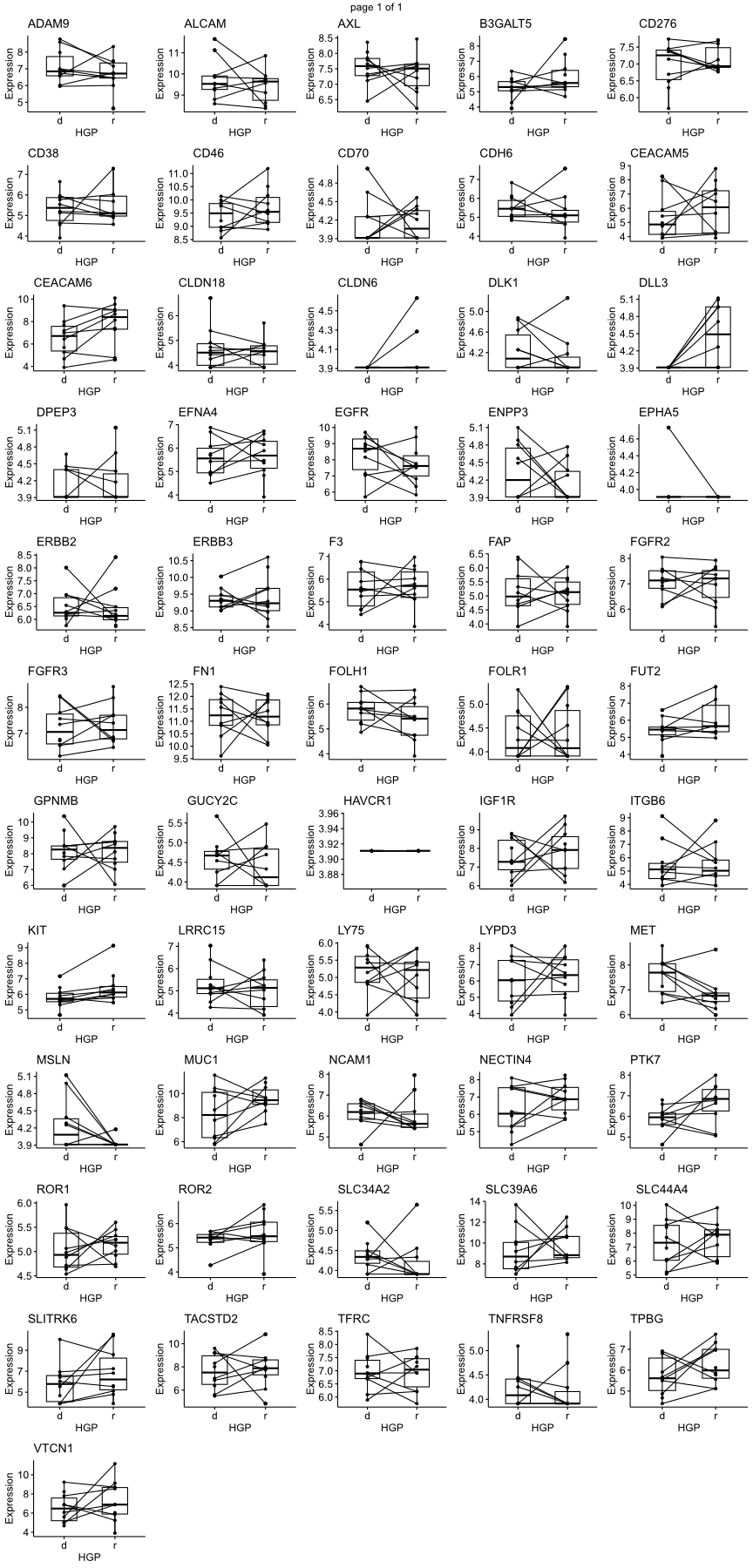


**Supplementary Figure 9: Normalized expression of the 56 ADC genes according to HGP.** Abbreviations: r= replacement; d= desmoplastic; HGP= histopathological growth pattern.
