# Supplementary material for: Transcriptomic characterization of the histopathological growth patterns in breast cancer liver metastases
Source: Clin Exp Metastasis. 2024 Mar 29;41(5):699–705. doi: 10.1007/s10585-024-10279-1 (PMC11499425; doi:10.1007/s10585-024-10279-1)
Supplement: Supplementary file 4 — Supplementary Material 4 [file 10585_2024_10279_MOESM4_ESM.docx]

**Supplementary Material**

**Methods**

***RNA isolation, library preparation, sequencing and data processing***

RNA was extracted from recently cut up to 10 μmx4 FFPE sections of breast cancer liver metastases, without microdissection, according to the QIAGEN miRNeasy FFPE kit (ref#217504) instructions ^4^. The paraffin was removed from freshly cut FFPE tissue sections by using 320μl of the Deparaffinization Solution. The samples were placed in a 56°C heat block with 240μl of optimized lysis buffer (Buffer PKD) and 10μl of Proteinase K. This combination helps release RNA from the tissue sections. To improve the yield and quality of the RNA, as well as its performance in downstream enzymatic assays, a short incubation at 80°C was performed to partially reverse the formalin crosslinking of the released nucleic acids. DNase treatment was applied to eliminate all genomic DNA. This treatment involved using a mixture of 1:10 DNase Booster Buffer and 10μl of DNase I stock solution, which has been optimized for this purpose. Next, the lysate was combined with 500μl of Buffer RBC. To ensure proper binding conditions for RNA, 1750μl of ethanol was added. Subsequently, the sample was applied to a RNeasy MinElute spin column in multiple steps of 700μl until the whole sample passed through, where the total RNA binds to the membrane while contaminants were effectively washed away using two rounds of 500 μl of Buffer RPE. Finally, the RNA was eluted using a minimum of 25μl of RNase-free water, which allowed for its release from the column. Extracted RNA was quantified using NanoDrop^TM^ One spectrophotometer. The extracted RNA was subsequently sequenced using the lexogen protocol (3’ mRNA FWD Quantseq). The RNA reads were mapped to the GRCh38 reference genome using the STAR aligner (v2.7.10a). Genes were quantified using the subread package (v2.0.3), followed by normalization of gene counts using the variance stabilizing transformation approached implemented in the DESeq2^5^ package (v1.32.0).

***Tumor Cellularity assessment***

The tumor cellularity was assessed by a pathologist (G.Z.) for each sample as the proportion (%) of tumor cells on the entire section.

***Histopathological growth pattern assessment***

The HGP were assessed on all H&E-stained slides according to the international consensus guidelines by one independent pathologist (P.V.) using light microscopy[11, 13]. For each patient, the HGP was scored for all the slides available of all the metastases as a relative proportion (percentage) of each HGP at the tumor-liver interface. We selected 10 patients for whom at least two samples presented different HGP, replacement (r-HGP) or desmoplastic (d-HG), allowing us to avoid the bias related to patient heterogeneity. The two main growth patterns were present within the same metastasis (i.e. one FFPE sample presenting mainly an r-HGP component and another sample mainly a d-HGP component at the tumor-liver interface). A minimum of 40% of one of the HGP was used as the threshold to select the patients and the H&E slides and this cut-off was used to define the HGP of the sample, as one sample was replacement-type if more than 40% of its tumor-liver interface was replacement and same for desmoplastic.

***Tumor-infiltrating lymphocytes assessment***

We used the new guidelines defined in our previous paper[14] for scoring tumor-infiltrating lymphocytes (TIL) in breast cancer liver metastases. This modification allows TIL scoring in BCLM both on glass slides as well as on digitalized whole slides images of H&E sections. Five representative fields with vital carcinoma and adjacent liver parenchyma were evaluated for each slide, from which the average TIL score was derived. For each field, the respective growth pattern was noted. The scoring was performed at the interface between metastatic BC and liver (i.e. outer margin) using a 20x objective on standard microscopy, or a digital field with the major side of 800-1000 µm in length. For r-HGP, the scoring area was obtained by defining an imaginary line joining the two most outer cancer cells at the invasive front that touched (or crossed) the upper margin of the field of view. Thus, all TIL present in the tissue below this line were included in the scoring. For the d-HGP, the scoring area was found below the imaginary tangential line passing through the most outer point of the desmoplastic rim (which, importantly, included lymphocytic infiltration, when present). Small portal tracts falling below the imaginary outer margin of the BCLM were included but pre-existing fibrous structures/capsules were excluded as well as large blood vessels and necrotic areas. The TIL score (%) indicates the relative surface area of the non-epithelial component of the metastasis covered by lymphocytes.

***RNA isolation, library preparation, sequencing and data processing***

RNA was extracted from recently cut up to 10 μmx4 FFPE sections of breast cancer liver metastases, without microdissection, according to the QIAGEN miRNeasy FFPE kit (ref#217504) instructions [15] (Supplementary Material – Methods: ‘*RNA isolation, library preparation, sequencing and data processing’ section*). The extracted RNA was subsequently sequenced using the lexogen protocol (3’ mRNA FWD Quantseq). The RNA reads were mapped to the GRCh38 reference genome using the STAR aligner (v2.7.10a). Genes were quantified using the subread package (v2.0.3), followed by normalization of gene counts using the variance stabilizing transformation approached implemented in the DESeq2[16] package (v1.32.0).

***Cell deconvolution***

Computational inference of the quantitative proportions of 64 cell types in each sample was performed using xCell [17] (<http://xCell.ucsf.edu/>) from the normalized gene expression matrix. xCELL is a marker gene approach to identify enrichment of specific cell types present within a sample. xCell uses a single gene set enrichment approach (ssGSEA or GSVA) and it uses a cohort of 489 gene sets designed to represent the transcriptomic composition of 64 different cell types coming from 5 different cell families (lymphoid cells, myeloid cells, stromal cells, stem cells and cells of origin). It also includes an immune and microenvironment score (MES), representing the total abundance of immune and stromal cell types in each sample was computed by xCell and used in subsequent analyses. The enrichment scores of each cell types are summarized in Supplementary Table S6. The associations between the immune cells and the HGP were estimated by linear mixed models adjusted for the Cellularity and the Microenvironment Score MES. Cell types with a p-value < 0.1 in one of the two models are shown in Figure 2a and cell types with a p-value < 0.2 in one of the two models are shown in Supplementary Fig 5. P-values presented were not formally corrected for multiple testing. Desmoplastic HGP was used as the baseline/reference for the differential analyses.

***Differential gene expression analysis (DGEA)***

Differential gene expression according to HGP was performed on the raw gene count matrix using the DESeq2 package (v1.32.0). A model matrix was defined with HGP, patient, and either pathologically assessed tumor cellularity or computationally derived MES, as covariates. We used the PANTHER (Protein Analysis Through Evolutionnary Relationship) Classification system to classify the differentially expressed genes (p-value < 0.1) based on their functions[18, 19]. REVIGO^7^, a web tool that summarizes long, unintelligible lists of GO terms by finding a representative subset of the terms using a clustering algorithm that relies on semantic similarity measures[20], was then used to facilitate interpretation and visualize the results. Given the exploratory nature and limited sample size of this study, p-values presented were not corrected for multiple testing. Desmoplastic HGP was used as the baseline/reference for the differential analyses.

***Gene Set Enrichment Analysis (GSEA)***

We performed gene set enrichment analysis using the unsupervised single sample-based method Gene Set Variation Analysis (R package ‘GSVA’— v1.40.1)[21]. GSVA scores of gene sets in the C5:GO:BP collection retrieved from the MSigDB database (v7.5.1) were calculated for each sample from the normalized gene expression matrix. Desmoplastic HGP was used as the baseline/reference for the differential analyses.

***Statistical analyses***

Statistical analyses were performed using R version 4.1.1. The association between HGP and the enrichment of each gene set was then assessed using linear mixed models, with the HGP and either tumor cellularity or MES as fixed effects, and patient as the random effect. Similar analyses were performed to evaluate the association between HGP and the deconvolution-derived cell proportions. P-values presented were not corrected for multiple testing. Significance of the association between the HGP and TIL is assessed by a Wilcoxon-Man Whitney test.

**References**

1. Van Dam, P. J. *et al.* International consensus guidelines for scoring the histopathological growth patterns of liver metastasis. *Br J Cancer* **117**, 1427–1441 (2017).

2. Latacz, E. *et al.* Histopathological growth patterns of liver metastasis: updated consensus guidelines for pattern scoring, perspectives and recent mechanistic insights. *British Journal of Cancer 2022 127:6* **127**, 988–1013 (2022).

3. Leduc, S. *et al.* Histopathological growth patterns and tumor-infiltrating lymphocytes in breast cancer liver metastases. *NPJ Breast Cancer* **9**, 100 (2023).

4. Qiagen. *miRNeasy FFPE Handbook*. (2018).

5. Love, M. I., Huber, W. & Anders, S. Moderated estimation of fold change and dispersion for RNA-seq data with DESeq2. *Genome Biol* **15**, 1–21 (2014).

6. Aran, D., Hu, Z. & Butte, A. J. xCell: Digitally portraying the tissue cellular heterogeneity landscape. *Genome Biol* **18**, 1–14 (2017).

7. Thomas, P. D. *et al.* PANTHER: Making genome-scale phylogenetics accessible to all. *Protein Science* **31**, 8–22 (2022).

8. Mi, H., Muruganujan, A. & Thomas, P. D. PANTHER in 2013: modeling the evolution of gene function, and other gene attributes, in the context of phylogenetic trees. *Nucleic Acids Res* **41**, (2013).

9. Supek, F., Bošnjak, M., ˇ Kunca, S. & ˇ Muc, S. REVIGO Summarizes and Visualizes Long Lists of Gene Ontology Terms. *PLoS One* **6**, 21800 (2011).

10. Hänzelmann, S., Castelo, R. & Guinney, J. GSVA: Gene set variation analysis for microarray and RNA-Seq data. *BMC Bioinformatics* **14**, 1–15 (2013).
